# Toward Subcellular Action Potential Detection with Nanodiamond Quantum Magnetometry

**DOI:** 10.3390/nano15241879

**Published:** 2025-12-15

**Authors:** Azmath Fathima, Peker Milas, Sheikh Mahtab, Tanmay Talukder, Mya Merritt, James Wachira, Solomon Tadesse, Michael Spencer, Birol Ozturk

**Affiliations:** 1 Department of Physics and Engineering Physics, Morgan State University, Baltimore, MD 21251, USA; peker.milas@morgan.edu (P.M.); shmah1@morgan.edu (S.M.); tatal3@morgan.edu (T.T.); mymer1@morgan.edu (M.M.); 2 Department of Biology, Morgan State University, Baltimore, MD 21251, USA; james.wachira@morgan.edu; 3 Center for Research and Education in Microelectronics, Morgan State University, Baltimore, MD 21251, USA; michael.spencer@morgan.edu; 4 Department of Chemistry, Morgan State University, Baltimore, MD 21251, USA; solomon.tadesse@morgan.edu; 5 Department of Electrical and Computer Engineering, Morgan State University, Baltimore, MD 21251, USA; 6 Department of Electrical and Computer Engineering, Cornell University, Ithaca, NY 14850, USA

**Keywords:** NV diamond, quantum sensing, action potentials, ODMR

## Abstract

Quantum sensing with nitrogen vacancy (NV) defects in diamond enables detection of extremely small changes in temperature, host material strain, and magnetic and electric fields. Action potential detection has previously been demonstrated with cardiac tissue and whole organisms using NV defects in bulk diamond crystals. Nanodiamonds (NDs) with NV defects were previously used as effective fluorescent markers, as they do not bleach under laser illumination like conventional fluorescent dyes. Subcellular-level action potential recording with NDs is yet to be demonstrated. Here, we report our results on the confocal imaging of NDs and the feasibility of optically detected magnetic resonance (ODMR) experiments with Cath.-a-differentiated (CAD) mouse brain cells. 10 nm and 60 nm NDs were shown to diffuse into cells within 30 min with no additional surface modification, as confirmed with confocal imaging. In contrast, 100 nm and 140 nm NDs were observed to remain localized on the cell surface. ND photoluminescence (PL) signals did not bleach over the course of 5 h long imaging studies. ODMR technique was used to detect externally applied millitesla-level magnetic fields with NDs in cell solutions. In summary, NDs were shown to be effective, non-bleaching fluorescent markers in mouse brain cells, with further potential for use in action potential recording at the subcellular level.

## 1. Introduction

Nanodiamonds (NDs) with NV defects offer distinct advantages over traditional dyes and quantum dots. Their exceptional attributes include high quantum yield, minimal toxicity, and extreme photo and thermal stability [1,2,3,4,5,6,7,8,9,10,11,12,13,14,15]. Notably, NDs also find applications in wide-field spin-based ND thermometry in intracellular temperature measurements of live organisms [16,17,18,19,20,21,22,23,24].

Moreover, quantum magnetometry using nitrogen vacancy (NV) color center defects in bulk diamond and NDs is an emerging paradigm-shifting technology [25,26]. Local magnetometry is a fundamental method of characterizing nano- and micro-molecules using a wide range of scanning techniques, including NV defects in diamonds. It provides magnetic field mapping at the nanometer scale and is frequently utilized in material characterization. Current local magnetometry techniques for contactless recording of action potentials rely on the detection of ion current-induced magnetic fields. As in the case of magnetoencephalography (MEG), these techniques are very costly, the necessary instruments are bulky, and they all require cryogenic cooling. On the other hand, existing action potential recording methods with contacts at the single-cell level are laborious and invasive.

Quantum sensing of action potentials with local magnetometry in cardiac tissue and in whole organisms has been previously demonstrated using NV defects in bulk diamond at room temperature [27,28,29,30]. Yet, the realization of action potential recording from individual cells with nanoscale spatial resolution and sub-millisecond temporal resolution using NDs remains an unexplored frontier. This innovative approach not only holds promise for high-resolution cellular-level recording but also enables the possibility of intracellular action potential monitoring through cell uptake of NDs. Consequently, NDs emerge as versatile probes capable of providing unprecedented insights into intracellular dynamics.

Globally, dementia affects about 57 million people, with nearly 10 million new cases each year, and over 60% of those affected living in low- and middle-income countries [31]. Alzheimer’s disease accounts for roughly 60–70% of all cases and contributes to dementia being the seventh leading cause of death worldwide, imposing an annual economic burden of about USD 1.3 trillion [31,32]. In the absence of a universal cure, the World Alzheimer Report 2023 stresses risk reduction measures such as maintaining cardiovascular health, social engagement, and lifelong learning [32].

Likewise, cardiovascular diseases (CVDs) remain the leading cause of death, responsible for about 19.8 million deaths in 2022, or 32% of all global deaths, with 85% due to heart attack or stroke [33]. These figures underscore the critical need for ultrasensitive, non-invasive detection technologies that can capture early electrophysiological signals. Nanodiamond-based quantum magnetometry offers such potential, enabling subcellular-scale magnetic field sensing for early prognosis of neurodegenerative and cardiac disorders.

Furthermore, recent advances highlight the importance of biological magnetic nanomaterials, which exhibit unique magneto-biological properties useful in biosensing, drug delivery, and imaging applications [34]. Nitrogen vacancy (NV) quantum magnetometry offers a highly sensitive, non-invasive approach to characterize such materials under physiological conditions with nanoscale spatial resolution [35]. Incorporating these developments emphasizes the broader impact of NV-based nanodiamonds quantum sensing in studying magnetic biomaterials and cellular bioelectromagnetic phenomena.

Here, we present our results on the potential use of NDs in subcellular-level action potential recording from neuron cells. We first studied uptake of different size NDs in CAD mouse brain cells. We observed optically detected magnetic resonance (ODMR) from NDs in the presence of live mouse brain cells, a critical step towards recording high-spatial resolution action potentials from induced magnetic field signals. Externally applied millitesla-level magnetic fields generated by a Helmholtz coil were measured with ND quantum sensing. We also describe the required conditions to successfully obtain the reported results and discuss future developments needed for successful utilization of NDs in intracellular action potential detection from neuron and cardiac cells.

## 2. Materials and Methods

Cell culture—Cath.-a-differentiated (CAD) cells (provided by Dr. James Wang, Cogent Neuroscience Inc., Durham, NC, USA) were cultured according to standard culture procedures [36]. CAD cells were grown in a mixture of Dulbecco’s Modified Eagle Medium (DMEM) and F-12 medium in a 1:1 ratio, both of which are commonly used for maintaining neuronal cells due to their balanced nutrient profile. The medium was supplemented with 8% fetal bovine serum (FBS) to provide necessary growth factors, as well as 1% penicillin–streptomycin to prevent bacterial contamination, ensuring a sterile environment for optimal cell health. The cells were cultured in cell culture dishes and kept in a controlled environment in a humidified incubator maintained at 37 °C with 5% CO_2_ to replicate physiological conditions for cell growth.

Routine passaging of the cells was performed every two days to prevent overcrowding, which could compromise cell health and experimental reproducibility. The cells were carefully detached by gentle pipetting to avoid damage and triturated into a fresh 5 mL medium to facilitate resuspension. Afterward, the cells were transferred into new culture dishes to continue their growth and division. This process allowed for the continuous propagation of healthy cells for experimental use.

As shown in the confocal images in the results section, representative confocal/DIC images show healthy CAD cell morphology with local confluency varying between 30 and 70% (average 28.7%), consistent with actively proliferating cultures prior to ND incubation.

NDs are of particular interest because their NV centers exhibit unique optical properties that can be utilized in various sensing and imaging applications. The NDs selected for this study had diameters of 10 nm, 60 nm, 100 nm, and 140 nm, offering a range of sizes to explore how particle size affects cellular uptake, interaction, and fluorescent behavior. The NDs were carefully suspended in the culture medium to ensure even distribution and facilitate their internalization by the CAD cells. This step allowed the cells to load the NDs efficiently for subsequent imaging and analysis. As-received ND solutions were diluted 10 times (1:9 ratio) with deionized (DI) water. Each batch of cells with nanodiamonds was incubated overnight with nanodiamonds. A Leica STELLARIS 5 (Leica Microsystems, Deerfield, IL, USA) inverted confocal microscope was used to obtain fluorescent images of the cells loaded with NDs.

**Figure 1:**
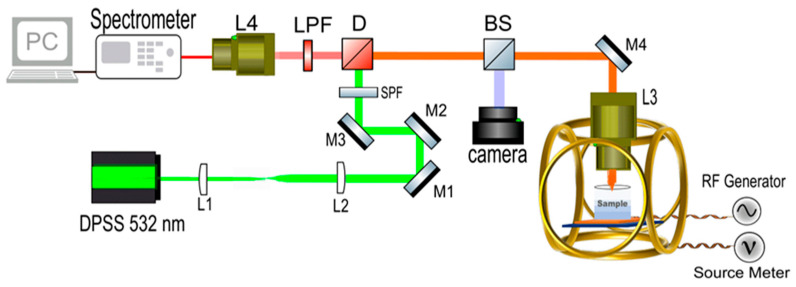
Schematic illustration of the experimental setup.

A custom-built confocal photoluminescence setup was employed in the room-temperature ODMR experiments, with a schematic depicted in Figure 1. The SecureSeal™ hybridization chambers (Grace Bio-Labs, Bend, OR, USA) containing the cells and ND solution were positioned on top of a custom-fabricated double-ring coplanar waveguide (CPW) antenna, between the ring and the feedline, where the antenna’s magnetic field intensity is at its maximum, based on computational simulation results. The return loss (S11) of the RF antenna was over −20 dB at 2.87 GHz, the ZFS frequency of negatively charged NV defects in diamond. The RF antenna’s detailed specifications were previously reported [25]. The bulk diamond (DNV-B14) used in the ODMR experiments was purchased from Element Six (Didcot, UK). A 532 nm diode-pumped solid-state (DPSS) laser was utilized to excite NV defects, while an HP Agilent 8648C RF signal generator (Santa Clara, CA, USA) was employed to apply microwaves to the sample via the RF antenna. PL signal was collected with an ASEQ Instruments HR1 CCD spectrometer (Vancouver, BC, Canada). A Newport 1830C power meter (Irvine, CA, USA) was employed to check laser power fluctuations. ODMR scans were performed in continuous-wave (CW) mode, where the excitation laser, microwave source, and PL spectra collection were always on during the measurements. A custom Python program was developed to control and synchronize all the instruments during experiments, which also collects and post-processes the data. This program starts the RF sweep and records the NV-diamond PL emission intensity at each microwave frequency to generate the ODMR data. In all ODMR experiments, the RF sweep step size was 1 MHz and the sweep range was from 2800 MHz to 3000 MHz. For clarity purposes, varying partial sweep ranges are shown in Section 3. Microwave power at the sample was 20 dBm. ODMR scans commenced only after laser power stabilizes, with fluctuations below 1%. Background noise counts of the spectrometer were subtracted using expectation–maximization (E-M)-based machine learning (ML) algorithms. These ML algorithms effectively clean up the spectra by eliminating initial noise, resulting in precise PL data collection with the spectrometer.

## 3. Results

**Figure 2:**
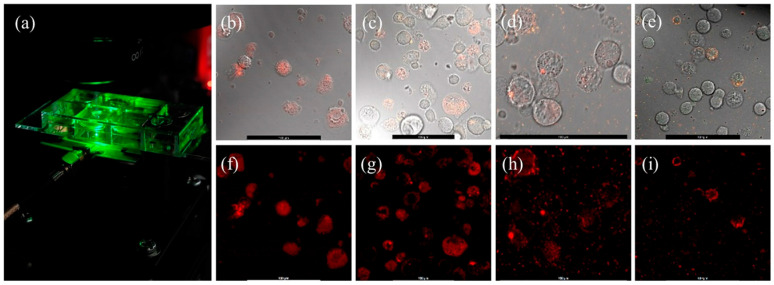
(**a**) Sample-holder slide with eight wells. (**b**–**e**) DIC images with laser overlap from CAD mouse neuron cells loaded with 10 nm, 60 nm, 100 nm, and 140 nm diameter NDs. (**f**–**i**) Corresponding confocal PL images with a 568 ALEXA laser. Scale bars on all images represent 100 μm.

We deposited different sizes (10, 60, 100, and 140 nm) of NDs in the growth medium of CAD mouse brain cells and performed confocal PL microscope imaging, which revealed the uptake of NDs by the cells. NDs were deposited in the growth medium at the beginning of cell culture and incubated overnight before confocal PL imaging. Control samples were prepared with only CAD cells and with only NDs, and they were also tested with confocal PL imaging. Cells and NDs were cultured in slides with eight wells as shown in Figure 2a. Each of the eight wells could hold 300 μL of solution. Cells were directly cultured in the slide wells overnight so that they can adhere to the bottom surface. This approach facilitated imaging as an inverted confocal microscope was used with a 63x oil immersion objective. Confocal imaging experiments were several hours long, and no indication of bleaching was observed in ND PL signals. Figure 2b–i show differential interference contrast (DIC) and corresponding PL images of cells with different-sized NDs. The cells mostly took up 10 nm NDs via endocytosis. The cells also took up 60 nm NDs, but at a slightly lower rate, with some NDs appearing to be attached to the outer surfaces of the cells. The outer surfaces of cells were mostly occupied by 100 nm NDs. Only some of the 140 nm NDs were attached to the outer surfaces of cells, and most of them remained in the solution.

**Figure 3:**
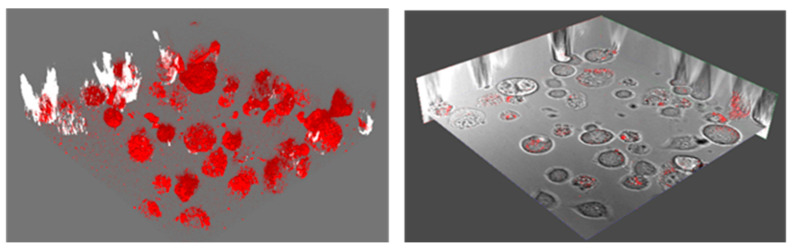
Confocal 3D PL emission from 60 nm NDs in live CAD cells and a layer of the corresponding cross section DIC image, showing the localization of the PL signals to cells only.

Figure 3 depicts the 3D PL and the corresponding cross-sectional DIC images obtained from Z–stack confocal microscopy of neuron cells incubated with 60 nm NDs overnight. PL signals were mostly localized in cell locations, and the cross-sectional DIC image shows that 60 nm diameter NDs were absorbed by some of the cells. In future experiments, micropipettes will be used to deposit NDs directly into a targeted cell, which will enable ND density control in individual cells.

**Figure 4:**
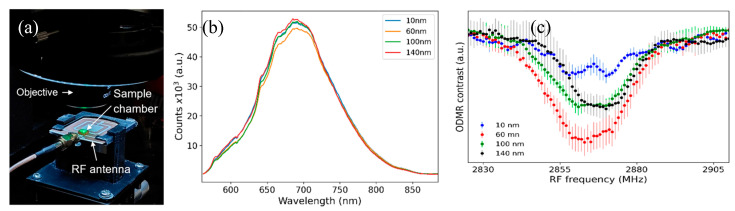
(**a**) An image of the confocal PL sample stage, showing the RF antenna and the SecureSeal™ hybridization chamber sample holder with NDs and live cells. (**b**) PL signal from ND samples in cells. (**c**) Zero (magnetic)-field averaged ODMR spectra obtained with different-sized NDs absorbed by cells.

We have conducted a preliminary study to demonstrate the feasibility of our approach in action potential recording by quantum sensing of induced magnetic fields. We initially performed ODMR experiments with eight well slides containing NDs and cells. As depicted in Figure 2, the radio frequency (RF) antenna is at the bottom of the sample, and the signal must be collected from the bottom of the wells for the microwaves generated by the antenna to efficiently interact with the NDs. Due to the extra volume (300 μL) of growth medium used in the wells, laser and PL emission were diffracted, and we could not perform ODMR scans. Thus, we placed ND and cell solutions in 4 mm diameter SecureSeal™ hybridization chambers with 25 μL volume for ODMR measurements, as shown in Figure 4a. In these sample holders, ODMR results without any applied magnetic field were successfully obtained with NDs in live CAD cells, demonstrating the potential of NDs to detect magnetic fields induced by action potentials in live cells. Representative PL spectra for all NDs are shown in Figure 4b.

Integration times were adjusted to obtain maximum count rates before ODMR experiments. The average of several zero-field ODMR scans for each ND size is shown in Figure 4c. These data were obtained from NDs absorbed by the CAD cells.

The triplet electronic spin Hamiltonian of the NV center in the presence of an external magnetic field (*B*) is given as follows

(1) $$H=hDS_{z}^{2}+h\gamma_{e}BS_{z}$$

where *h* is the Planck’s constant, *S_z_* is the spin operator, *D* is the ZFS value, and $\gamma_{e}$ is the gyromagnetic ratio (28 MHz/mT) of the NV spin. Although there was no applied magnetic field, splitting or broadening is visible in all the measured ODMR spectra due to hyperfine interactions between the NV center electron spins and the nitrogen nuclear spins, which split the resonance into multiple closely spaced frequencies. ODMR spectra were recorded in the presence of adult mouse brain cells, and no stimulation was applied to the cells to induce action potentials. The magnitude of the action potential-induced magnetic field was previously measured as 4 nT peak-to-peak with bulk diamond NV defects from an excised individual marine worm neuron [29]. The expected ODMR splitting or shift is about 100 Hz for neuron action potential-induced magnetic fields, which cannot cause the observed splitting of the ODMR spectra with no externally applied magnetic field. This estimate also shows that hertz-level RF sweep resolution is needed in future experiments for action potential detection with NDs from individual neuron cells.

**Figure 5:**
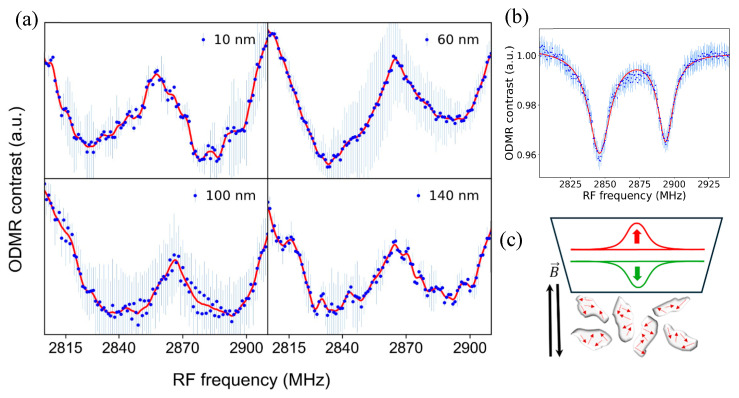
(**a**) Detection of externally applied magnetic field with NDs in live cells. For all ND sizes, the ODMR dips split for about 50 MHz, corresponding to the applied 1.7 mT field. (**b**) ODMR magnetic field sensing spectra obtained with bulk diamond, showing that the ODMR dips are much narrower and well defined compared to ND ODMR spectra. (**c**) A schematic showing the random orientations of NDs and the NV defects (red arrows), which causes broadening of the ODMR dips.

The magnetic field sensing capability of NDs in cells was tested by performing ODMR scans with externally applied 1.7 mT magnetic field to the samples with a Helmholtz coil. ODMR scan results are shown in Figure 5a, where the blue dots are collected data points and represent averages of two or three measurements. While bulk diamond data are typically represented using a double-Lorentzian model, the ODMR data of NV nanodiamonds present a challenge as they cannot be adequately modeled as a sum of two functions. Consequently, we employed cubic spline interpolation on the data after applying smoothing, as represented by the continuous red lines. About 50 MHz splitting of the ODMR dips are visible for all ND sizes due to the Zeeman effect, which agrees with the gyromagnetic ratio of NV defects as shown in Equation (1).

Figure 5b shows the 1.7 mT magnetic field detection ODMR spectra with a bulk diamond sample, where the applied single-axis magnetic field by the Helmholtz coil was parallel to the Z-axis of the bulk diamond sample. A comparison of the ND and bulk diamond ODMR spectra shows that the ND ODMR spectra dips are much broader compared to bulk diamond. This broadening is due to random orientations of the ND samples in the cell solution as depicted in Figure 5c. Since the angle between the NV axis and the external magnetic field varies across NDs, the Zeeman splitting differs, leading to a distribution of resonance frequencies. This results in the broadening of the sharp ODMR dips observed in bulk crystals with aligned NV centers (Figure 5b). As shown in Figure 2 and Figure 3, high densities of NDs were present in the cells. A 50x long-working-distance objective with 0.42 numerical aperture (NA) was utilized for confocal PL signal collection from an area with approximately 10-micron diameter. Thus, the collected PL signal was from ensembles of NDs with different orientations. Each ND hosts multiple negatively charged NV defects with random orientations, causing broadening of the ODMR dips due to the averaging of signals while interacting with the external magnetic field.

It should be noted that while ODMR and magnetic field sensing experiments were conducted in the presence of live CAD cells, these measurements did not target magnetic fields generated by the cells themselves. Instead, the purpose was to establish the feasibility of performing stable ODMR in biological environments containing live cells and nanodiamonds. Future work will focus on detecting cell-induced magnetic fields associated with neuronal action potentials using enhanced ODMR sensitivity.

### Strategies for Future Realization of Action Potential Detection from Individual Cells Using NDs

Natural uptake of NDs by CAD cells was observed, but deposition of NDs into individual cells will be performed in future experiments. To perform these targeted individual cell studies, micropipettes and a nanoliter dispenser will be used. We are currently optimizing our custom-built confocal PL setups using machine learning algorithms to demonstrate detection of picotesla-level magnetic field signals. An important aspect of action potential detection is the capability of measuring vectorial components of the induced magnetic fields. NV-diamond approach is inherently capable of performing vector magnetometry [37]. ODMR spectra displays signatures of each component of the applied vectorial magnetic field when fast photodiodes are utilized [29]. We have used CCD spectrometers in this project, which caused broadening of features in the ODMR spectra, as shown in Figure 5a. In future experiments, fast photodiodes will be utilized to capture the vectorial nature of the action potential-induced magnetic fields from live cells, which will provide insights into the details of intracellular action potential dynamics.

NV defects in lab-grown bulk diamonds have well defined crystallographic orientation, enabling their use in vector magnetometry applications. High-resolution ODMR spectra from single or ensemble NDs may provide vector magnetometry detection capability using machine learning algorithms. In future experiments, a nano volume injector will be used in the deposition of very dilute ND solutions into individual cells. Imaging will be performed using a 100x oil immersion lens for the maximum available NA to increase the collection efficiency of emission from individual NDs with low-density NV defects. These approaches will enable the collection of ODMR signals with narrower dips and well defined signatures of the vectorial components of action potential-induced magnetic field components.

## 4. Conclusions

This study demonstrates the feasibility of performing ODMR and magnetic field sensing measurements with NDs in live mouse brain cells. The 3D confocal imaging of the cells revealed size-dependent ND uptake by the cells. Room-temperature PL spectra were collected with a custom-built confocal PL setup. Optically detected magnetic resonance experiments were conducted with the NDs while they were in live mouse brain cell solutions. Externally applied millitesla-level magnetic fields were measured with the NDs in live cells using the ODMR method. Our results show the potential of this ND-based quantum sensing approach for recording action potentials from individual neuron cells through the detection of induced magnetic fields.

Future research will focus on enhancing the magnetic sensitivity and spectral resolution of NV-based nanodiamond probes to achieve direct detection of action potential-induced magnetic fields from individual neurons. This will involve integrating pulsed-ODMR-based magnetic field detection approaches such as the Ramsey sequence, single-cell ND deposition using micropipettes, and machine learning-assisted spectral analysis for improved signal extraction. Extending this approach to cardiac and neuronal tissues will further enable quantitative mapping of bioelectric activity with subcellular precision, advancing the development of non-invasive quantum biosensing platforms for biomedical diagnostics.

## Data Availability

The original data presented in the study are openly available in the repository ZENODO at DOI: https://doi.org/10.5281/zenodo.16326563 and reference number 16326563.

## Abbreviations

The following abbreviations are used in this manuscript:

ODMR	Optically Detected Magnetic Resonance
NV	Nitrogen Vacancy
NDs	Nanodiamonds
CAD	Cath.-a-differentiated
ML	Machine Learning
